# Dose-Dependent Effects of a Corn Starch-Based Bioplastic on Basil (*Ocimum basilicum* L.): Implications for Growth, Biochemical Parameters, and Nutrient Content

**DOI:** 10.3390/toxics12010080

**Published:** 2024-01-17

**Authors:** Nazanin Azarnejad, Silvia Celletti, Majid Ghorbani, Riccardo Fedeli, Stefano Loppi

**Affiliations:** 1 Department of Life Sciences (DSV), University of Siena, 53100 Siena, Italy; n.azarnejad@student.unisi.it (N.A.); m.ghorbani@student.unisi.it (M.G.); riccardo.fedeli@student.unisi.it (R.F.); stefano.loppi@unisi.it (S.L.); 2 BioAgry Lab, University of Siena, 53100 Siena, Italy; 3 BAT Center—Interuniversity Center for Studies on Bioinspired Agro-Environmental Technology, University of Naples “Federico II”, 80138 Napoli, Italy

**Keywords:** antioxidants, biomaterials, ecotoxicological risk, malondialdehyde, mineral elements, proline

## Abstract

Plastic pollution is a pressing global issue, prompting the exploration of sustainable alternatives such as bioplastics (BPs). In agriculture, BPs have gained relevance as mulching films. This study investigated the effect of the presence in the soil of different concentrations (0–3%, *w*/*w*) of a corn starch-based bioplastic on basil (*Ocimum basilicum* L.). The results showed that increasing bioplastic concentration reduced shoot fresh biomass production. Biochemical analyses revealed changes in the shoot in soluble protein content, biomarkers of oxidative and osmotic stress (malondialdehyde and proline, respectively), anti-radical activity, and antioxidant compounds (phenols, flavonoids, and ascorbic acid), which are indicative of plant adaptive mechanisms in response to stress caused by the presence of the different concentrations of bioplastic in the soil. Macro- and micronutrient analysis showed imbalances in nutrient uptake, with a decreased content of potassium, phosphorus, and manganese, and an increased content of magnesium, iron, and copper in the shoot at high BP concentrations.

## 1. Introduction

The massive presence of plastic waste, known for its persistence and adverse environmental impacts, has raised the need for eco-sustainable alternatives [1,2]. Bioplastic (BP), derived from natural renewable resources and/or exhibiting biodegradable properties, has attracted attention as a potential substitute of conventional plastic [3,4,5]. Bioplastics find applications in diverse sectors, including agriculture, where they are explored for their potential agronomic benefits and environmental implications [6,7,8,9]. In particular, the use of BP films covers various agricultural applications such as mulching, seed coating, and soil stabilization [10,11,12]. For instance, mulching films offer potential benefits such as an improved soil moisture retention and structure, and weed control, by contributing to enhanced crop yield but, most importantly, BP mulching films also limit the environmental impact, by reducing plastic waste and promoting sustainable farming practices [13,14,15,16]. However, there is growing concern about the possible negative environmental implications of the increasing use of bioplastics, as they appear to behave similarly to conventional fossil-based plastics. Indeed, it has been noted that bioplastics are sources of release of additives (potentially toxic chemicals), which are then degraded into smaller molecules, at the level of monomers and oligomers [17,18]. Moreover, this degradation phenomenon that bioplastics undergo would also appear to occur much faster than that of traditional plastics. Finally, bioplastics can bind various pollutants, thus posing a further threat to the environment due to possible ecotoxicological effects [19]. Nevertheless, there is a knowledge gap on the consequences of gradually increasing concentrations of BP in the soil on crop plant growth and productivity. The physiological and biochemical responses of cultivated plants to the presence of the residues, derived from the degradation of these biomaterials, are not sufficiently understood; moreover, these responses are variable, depending on the origin, composition, size, and thickness, as well as the concentration of BP in the soil [20]. On the one hand, some studies [21,22,23] have observed neutral effects of BP residues on plant growth; on the other hand, other studies [24,25,26,27,28] have observed negative effects. Therefore, a comprehensive overall examination of the possible environmental and human health consequences of the excessive use of BPs in agriculture is of paramount importance. Our prior investigations [27,28] showed that the presence of a corn starch-based BP in the soil, at a concentration of 2.5% (*w*/*w*), had adverse effects on basil plant growth. Based on those findings, the primary aim of this research was to identify the critical threshold at which this type of BP hinders plant growth.

## 2. Materials and Methods

### 2.1. Bioplastic Preparation

Starch, an abundant and inexpensive polysaccharide of natural origin, is among the most widely used materials from which BPs can be produced [29]. The BP used in this experiment was corn starch-based and obtained following the experimental protocols by de Azevedo et al. [30] and Shafqat et al. [31], as modified by Celletti et al. [27]. In brief, BP was obtained by mixing corn starch powder with distilled water in a 1:7 ratio (g:mL). Next, glycerol in equal amount to the starch and 2.5 mL of glacial acetic acid were added to the resulting dispersion. The milky mixture was continuously stirred, to prevent the formation of bubbles and lumps, and heated to 85 °C. The bioplastic was formed as soon as the consistency changed from liquid to solid and the colour changed from white to transparent. While it was hot, it was poured and spread with a glass rod on a glass plate to allow a film to form, which was allowed to solidify. It was first incubated in an oven for 1 h at 100 °C and then at room temperature for a week before being cut into small pieces (about 5 × 5 mm) that were added to the soil.

### 2.2. Experimental Design and Plant Growth

The experimental design consisted of seven different growing conditions with eight biological replicates each. A total of 56 pots (1 plant/pot) were filled with 80 g of a commercial growing substrate (VigorPlant Italia Srl, Lodi, Italy). The characteristics of the substrate were as follows: moisture content = 43%; porosity = 92%; pH = 5.30 ± 0.03; electrical conductivity = 1.12 ± 0.01 mS cm^−1^; cation exchange capacity = 56.9 ± 2.7 meq 100 g DW^−1^. The soil was supplemented with the corn starch-based bioplastic at the following concentrations (*w*/*w*): 0% (C, control), 0.5% (B0.5), 1% (B1), 1.5% (B1.5), 2% (B2), 2.5% (B2.5), and 3% (B3).

Basil (*Ocimum basilicum* L., cv. Riviera Ligure) seeds were germinated in the dark at 22 °C between several layers of soaked paper for five days. Then, the resulting seedlings were transplanted into pots (previously prepared as described above) and allowed to grow in a climate chamber with a temperature of 25/20 °C (day/night), a relative humidity of 70%, a photoperiod of 16/8 h (day/night), and a light intensity of 250 μmol m^−2^ s^−1^ PAR. Soil moisture was maintained at 70% of the water holding capacity by checking the weight of each pot every three days. The experimental plant growing period lasted 35 days. Afterwards, shoots of the plants were harvested, measured (see Section 2.3), and immediately stored at −20 °C for later analyses. The experiment was replicated three times under the same growing conditions.

### 2.3. Biometrics and Biochemical Parameters

At harvest, the number of leaves was recorded, and the height of the stem and the shoot fresh weight of the basil plants were measured.

The total soluble protein content was determined in the frozen shoots of basil plants by homogenizing 0.5 g of material in 3 mL of distilled water. After two centrifugations to obtain the sample, 20 µL of supernatant was diluted by adding 980 µL of distilled water. Subsequently, 0.4 mL of the diluted sample was added to 1.6 mL of Bradford reagent [32] and vigorously shaken. After 20 min of reaction, the sample absorbance was read at 595 nm using a UV–Vis spectrophotometer (8453, Agilent, Santa Clara, CA, USA). For the calculation of the protein content, bovine serum albumin was used as standard [33].

To determine the total antioxidant power, 0.2 g of frozen shoot tissue was homogenized with 2 mL of 80% (*v*/*v*) ethanol. After a centrifugation step at 4000 rpm for 5 min, 100 µL of supernatant was added to 1 mL of 2,2-diphenyl-1-picrylhydrazyl (DPPH) solution [3.9 mg of DPPH was dissolved in 100 mL of 80% (*v*/*v*) methanol]. A blank was prepared by adding 100 µL of 80% (*v*/*v*) ethanol into 1 mL of 80% (*v*/*v*) methanol and a control was prepared by adding 100 µL of 80% (*v*/*v*) ethanol into 1 mL of DPPH solution. The absorbance of the samples was measured at 517 nm using a UV–Vis spectrophotometer and the results were expressed as a percentage of anti-radical activity (ARA, %), according to the formula reported by Fedeli et al. [34].

The total contents of phenols and flavonoids were both determined using 0.3 g of air-dried shoots in the dark, according to the protocol of Borella et al. [35]. The samples were shaken with 3 mL of 80% (*v*/*v*) methanol for 30 min and then incubated for 48 h at 4 °C in the dark. The resulting extracts were filtered using Whatman filter paper no. 1. Phenols and flavonoids were quantified in the filtered samples using the Folin–Ciocalteu method [36] and an aluminium chloride colorimetric method [37], respectively. The absorbances of the samples were read at 760 and 415 nm, respectively, using an UV-Vis spectrophotometer. For the calculation of the content of phenols and flavonoids, gallic acid (5–300 µg mL^−1^) and quercetin (12.5–150 µg mL^−1^) were used as standards for the respective calibration curves.

The content of L-ascorbic acid (also known as vitamin C) was determined in the frozen shoots by following the method reported by Fedeli et al. [38]. After homogenizing, filtering, keeping on ice for 5 min, and finally centrifuging the samples, 0.4 mL of supernatant was vigorously mixed with 1.6 mL of distilled water and 0.2 mL of 2 M Folin–Ciocalteu reagent. After 10 min at room temperature, the absorbance of the samples was read at 760 nm using a UV-Vis spectrophotometer. For the calculation of the ascorbic acid content, ascorbic acid (5–20 µg mL^−1^) was used as standard for the calibration curve.

To determine the content of malondialdehyde (MDA, a biomarker of the level of cell plasma membrane lipid peroxidation), 0.5 g of frozen shoot material was homogenized in 5 mL of an extracting solution consisting of 0.25% (*w*/*v*) 2-thiobarbituric acid dissolved in 10% (*w*/*v*) trichloroacetic acid. The homogenates were heated at 95 °C for 30 min in a thermoblock and then rapidly cooled on ice to stop the reaction. Subsequently, the supernatants were collected by centrifuging the samples at 9300 rpm for 10 min. The absorbances were measured spectrophotometrically in the limpid supernatants at 532 and 600 nm. Calculations were made by subtracting the absorbance at 600 nm and using an extinction coefficient (155 mM^−1^ cm^−1^) of the formed MDA-TBA complex [39].

The content of proline was assessed following the protocols of Bates et al. [40] and Silvestri et al. [41]. Briefly, 0.1 g of frozen shoot material was homogenized in 2 mL of 5-sulfosalicylic acid dihydrate (3%, *w*/*v*). Then, the samples were centrifuged at 5000 rpm for 10 min, and 0.5 mL of supernatant was added to 0.5 mL of glacial acetic acid and 0.5 mL of acid-ninhydrin reagent (1.25 g of ninhydrin in 30 mL of glacial acetic acid and 20 mL of 6 M phosphoric acid). The mixtures were left to react for 1 h at 100 °C. Afterwards, the samples were placed on ice to block the reaction; finally, 1.5 mL of toluene was added to the samples. The content of proline was measured spectrophotometrically at 520 nm by reading the upper phase of the solution. For the calculation of the proline content, 1 mM L-proline was prepared as a stock solution and used as standard for the calibration curve (2–600 µL).

### 2.4. Nutrients

To analyze the content of macro- (calcium—Ca, potassium—K, magnesium—Mg, phosphorus—P, and sulfur—S), and micronutrients (copper—Cu, iron—Fe, manganese—Mn, sodium—Na, and zinc—Zn) in the shoots of basil plants, approximately 0.2 g of air-dried in the dark and pulverized material was acid-digested with 3 mL of 67% (*v*/*v*) nitric acid and 1 mL of 30% (*v*/*v*) hydrogen peroxide using a microwave lab station (Milestone Ethos 900, Shelton, CT, USA) as reported by Fedeli et al. [42]. After digestion, the samples were filtered and diluted with ultrapure water to a final volume of 50 mL. Inductively coupled plasma-mass spectrometry (ICP-MS, Perkin Elmer NexION 350, Waltham, MA, USA) was used to quantify the concentration of the above nutrients. The analytical quality was checked using the certified reference materials GBW 07604 (*Poplar leaves*) and GBW 07603 (*Branches leaves*), which showed recoveries in the range of 94–106%.

### 2.5. Statistical Analysis

The data approached a normal distribution (Shapiro–Wilk test, *p* < 0.05). Data are presented as the mean ± standard error of eight biological replicates. Statistical analysis was performed using one-way ANOVA and the least significant difference (LSD) test (*p* < 0.05) for post hoc comparisons. The R statistical software (version 4.3.2) [43] was used for all computations.

## 3. Results

### 3.1. Biometrics

**Figure 1:**
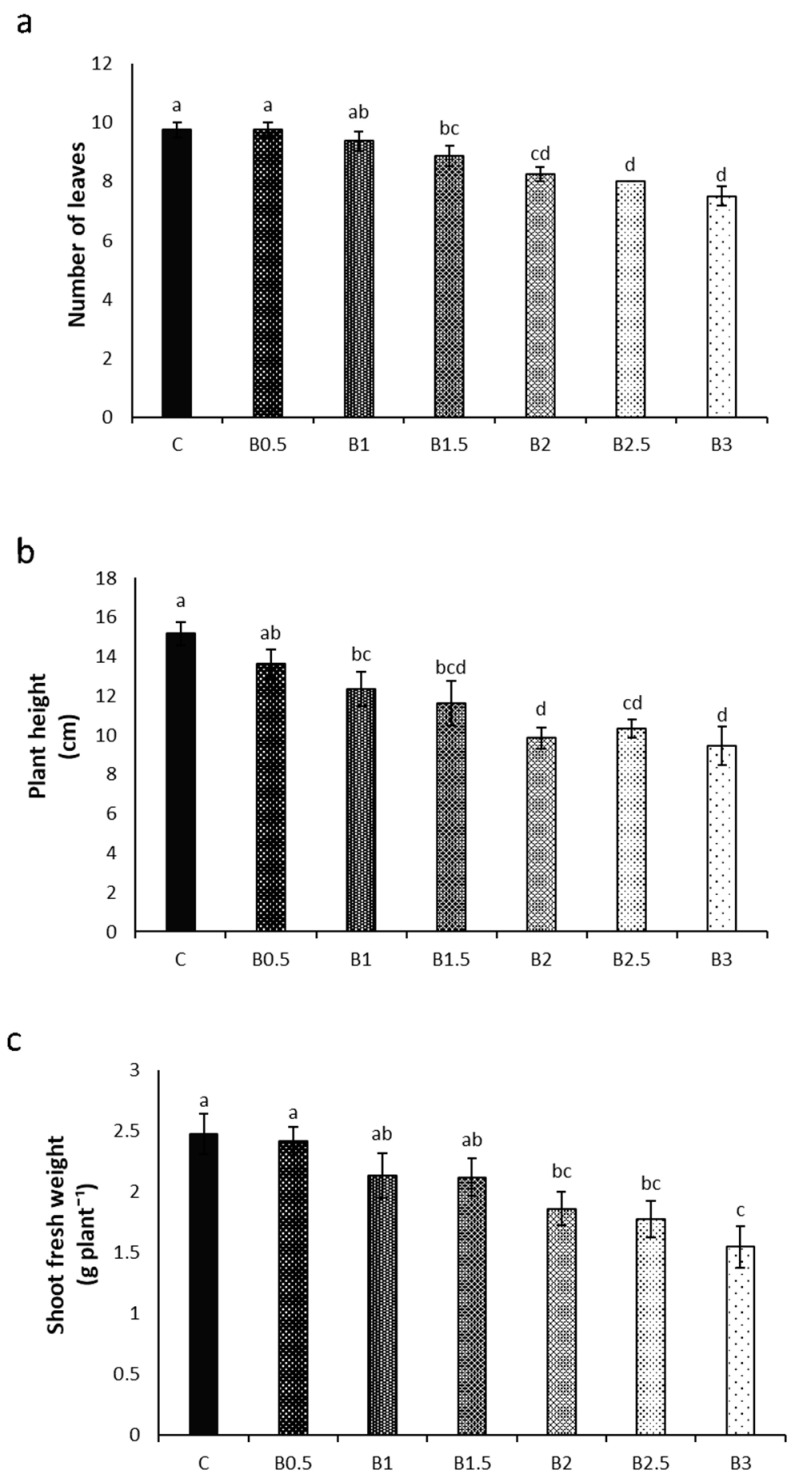
Effect of different corn starch-based BP concentrations [ranging from 0% (control = C) to 3%, *w*/*w*)] in the soil on the number of leaves (**a**), height (**b**), and shoot fresh weight (**c**) of basil plants. Different letters = statistically significant differences.

Control plants (C) showed the highest number of leaves (10/plant). Starting from B1.5, this parameter was reduced by 9% and then decreased gradually with increasing BP concentration; the lowest leaf formation was observed for B3 plants, which showed seven to eight leaves per plant (Figure 1a).

Similarly, plant height decreased gradually with increasing BP concentration: C plants reached a height of 15.2 cm, while B3 plants were the lowest, measuring on average 9.4 cm (Figure 1b).

Control plants also exhibited the highest shoot fresh weight (2.48 g), while an evident gradual decline of this parameter was observed with increasing BP concentration. Notably, B3 plants experienced a 37% reduction (Figure 1c).

### 3.2. Biochemical Parameters

**Figure 2:**
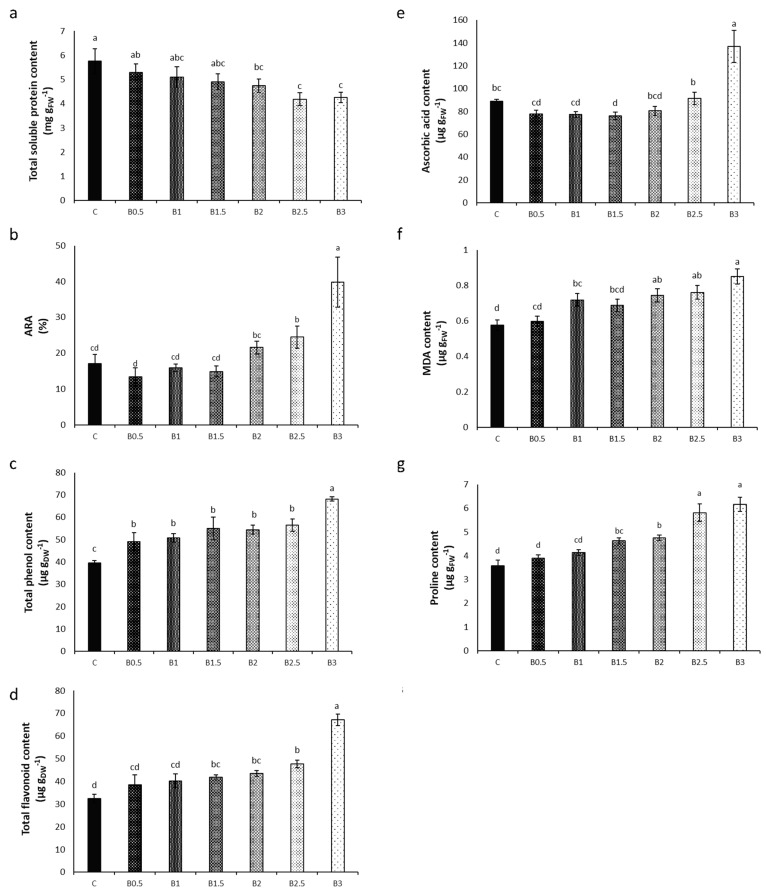
Effect of different corn starch-based BP concentrations [ranging from 0% (control = C) to 3%, *w*/*w*)] in the soil on the biochemical indexes determined in the shoot of basil plants. (a) Total soluble protein content; (**b**) anti-radical activity (ARA); (**c**) total phenol content; (**d**) total flavonoid content; (**e**) ascorbic acid content; (**f**) malondialdehyde (MDA) content; and (**g**) proline content. Different letters = statistically significant differences.

A gradual decline in the content of total soluble proteins in the shoot of basil plants with increasing BP concentration was evident. Control plants exhibited the highest (5.7 mg g_FW_^−1^) content, while B3 plants showed the lowest (4.3 mg g_FW_^−1^), corresponding to a reduction of 26% (Figure 2a).

No significant change in ARA was evident up to B2, while B2.5 and B3 plants showed a significant increase in ARA (+132% in B3) (Figure 2b).

B3 plants showed the highest contents of phenols and flavonoids, with increases of 72% and 106%, respectively (Figure 2c,d).

The content of ascorbic acid increased only in B3 plants (+54%) (Figure 2e).

The content of MDA, indicative of plant oxidative stress, was low in the shoot of C plants (0.6 µg g_FW_^−1^) and increased gradually with increasing BP concentration, reaching the highest value in B3 plants (+48%) (Figure 2f).

The content of proline, which is a bioindicator of osmotic stress, showed the highest value in B3 plants, with an increase of 72% (Figure 2g).

### 3.3. Nutrients

Among macronutrients, no statistically significant differences emerged for the content of Ca and S in the shoot of basil plants for all the different concentrations of BP tested. For Mg, a 20% increase emerged only in B3 plants. On the contrary, a significant reduction (−16%) in the content of K was observed only in B3 plants. Also, the content of P was reduced in B3 plants (−73%), with reductions being evident from B1.5 (Table 1).

Among micronutrients, no change emerged for Zn. Manganese was reduced by 50% already at B0.5. The content of Cu, Fe, and Na increased significantly (on average by 90%) only in B3 plants (Table 1).

## 4. Discussion

The present study aims to identify the critical threshold at which the adverse effects, caused by increasing concentrations of corn starch-based BP, occur on growth, biochemical parameters, and nutrients in the shoot of basil plants.

Our results showed notable negative effects of the investigated BP on key growth indicators, including the shoot fresh weight, height, and number of leaves of basil plants. Bioplastic concentrations > 1.5% (B1.5 growing condition) can hinder the plant’s capability to produce and accumulate the above-ground biomass. This reduction might be attributed to several factors, such as changes in nutrient availability or root development, potentially impairing the plant efficiency in nutrient uptake, or the hormonal balance within the plant [28].

Celletti et al. [27,28] showed that basil plants experienced a significant reduction in growth parameters when 2.5% corn starch-based BP was present in the soil. Similarly, Qi et al. [24] showed that wheat plants treated with 1% biodegradable macro- and micro- starch-based BP had a reduced vegetative growth. Brown et al. [20], Graf et al. [44], and Liu et al. [45] also mentioned the negative effects of biodegradable plastic on plant growth in maize. Serrano-Ruiz et al. [46] investigated different types of plastic and bioplastic mulches and showed a decrease in the amount of biomass and vegetative growth and a shrinking of the leaves in lettuce and tomato plants. Our results showed that BP size is not a driving factor, and the vegetative growth of the plant is reduced both using micro and macro particles. Rychter et al. [47] tested a tomato starch-based BP on horseradish and oat and found no significant effect on the growth of either crop. However, it should be noted that the BP concentration used in that experiment was five times lower (i.e., 0.1%) than the lowest concentration used in our experiment.

We observed a reduction in the content of total soluble proteins as BP concentration increased, suggesting a potential limitation in protein synthesis in basil plants. This decline in protein levels may be due to altered nutrient availability, limited root growth [28], or disruptions in metabolic processes induced by high BP concentrations, which could constrain basil’s capacity to produce proteins essential for growth and development. Furthermore, the increase in MDA content, indicative of the level of lipid peroxidation, and thus oxidative stress [48], in response to high BP concentrations, raises concerns about the potential oxidative damage caused by BP to basil plants. An elevated MDA content by increasing BP concentration suggests heightened vulnerability to oxidative stress, which could adversely affect the overall health and vitality of the basil plants. Similar results are reported by Gao et al. [49] on tomato seedlings, which showed an increase in MDA with increasing polystyrene nano-plastic concentrations, as well as Celletti et al. [27,28] on basil by testing 2.5% corn starch-based BP. In addition, the increase in proline in basil shoots implies that the presence of BP residues in the soil can induce an osmotic stress, potentially leading to plant adjustments to cope with unfavourable conditions. The higher the level of MDA and proline, the greater the damage to cell tissue, so that the plant activates its antioxidant response machinery to counteract oxidative stress [50]. Indeed, the rise in ARA, phenols, flavonoids, and ascorbic acid with increasing bioplastic concentration suggests a counteractive response by basil plants. The significant increase in ARA and ascorbic acid at BP concentrations exceeding 2% and 2.5%, respectively, suggests that basil plants may activate an adaptive antioxidant defence system to counteract reactive oxygen species (ROS) formation, probably generated under stress conditions at high BP concentrations [51]. To the best of our knowledge, although there is no extensive research on these parameters in the presence of BP residues in the soil, some studies have mentioned an increase in antioxidant compounds and secondary metabolites, including polyphenols and flavonoids, under stressful conditions [52,53,54]. The elevated ARA, along with increased phenol and flavonoid contents, reflects an enhanced capacity of the shoot of basil plants to counteract oxidative stress. Phenol and flavonoid compounds are well-documented antioxidants in plants, playing crucial roles in the detoxification of ROS [55]. Their accumulation in response to increasing BP concentration suggests that basil plants exploited these secondary metabolites as a strategy to mitigate the harmful effects of oxidative stress induced by BP residues. On the other hand, the increase in phenols and flavonoids with increasing BP concentration showed a similar trend with ascorbic acid as an antioxidant.

The presence of bioplastic in the soil is known to have a direct or indirect influence on the growth, biochemical parameters, and mineral content of crop plants [56]. Calcium and S content in the shoot of basil plants were unaffected across all BP concentrations. The increase in Mg, observed in the B3 plants, implies a potential adaptive response of basil plants to altered soil conditions induced by a high BP concentration. Indeed, a higher uptake of Mg, which is fundamental for the biosynthesis of the chlorophyll molecule and the photosynthetic process [57], may have occurred to compensate the environmental stress. However, the significant reduction in K in the B3 plants suggests that high BP concentrations could negatively affect the uptake of this important plant macronutrient, which is vital for enzyme activation and osmotic regulation [58]. The decrease in K may affect plant overall health and metabolic activities [59]. Phosphorus is one of the most important macronutrients and is necessary for plant growth [60]. The most striking observation was the substantial decrease in P in the B3 plants, suggesting a marked negative relationship between P uptake and high BP concentrations. Phosphorus is essential for energy transfer, nucleic acid synthesis, and ATP and DNA formation [61]. The drastic reduction in P content could have far-reaching implications for plant growth and development, potentially compromising various metabolic processes [62]. On the other hand, one of the properties of starch, which is the main component of our bioplastic, is to create adhesion between molecules [63]. According to this theory, it can be assumed that with the accumulation of BP in the soil, an increase in the adhesion of molecules in the soil occurred, and the plant could have lost the capability to absorb water and nutrients, especially P. In addition, the presence of biodegradable plastics in the soil could reduce the available P content in the rhizosphere by inhibiting soil enzyme activities [64,65]. Graf et al. [44] reported that the P content of maize shoots was decreased in the presence of high concentrations of macroplastic mulch residues, confirming the inhibition of nutrient uptake into the shoots at high concentrations.

Zinc content remained stable across all BP concentrations, suggesting that the plants were able to accumulate this essential micronutrient under all growing conditions. This resilience in Zn uptake may reflect the plant’s capability to meet its nutritional requirements even in the presence of BP. Manganese is crucial for various metabolic processes, including photosynthesis; a decrease in Mn accumulation could adversely affect these processes [66]. The significant reduction in Mn with increasing BP concentration indicates a sensitivity of Mn uptake to BP contamination. Therefore, it could be assumed that the bioavailability of Mn, whose content in the shoot showed the same trend as that of P, may be affected by the presence of BP, which binds Mn and, consequently, makes it less available for uptake by plants. The notable increase in Fe and Cu in the B3 plants suggests that high BP concentrations may influence the uptake of these micronutrients, with potential implications for chlorophyll synthesis, photosynthesis, and enzyme activities [67]. The consistent Na content across all BP concentrations, except for the B3 plants, suggests that BP did not induce significant alterations in Na accumulation within the basil plants up to a concentration of 2.5%.

## 5. Conclusions

Our study investigated the influence of increasing concentrations of corn starch-based BP in the soil on the growth, biochemical parameters, and nutrients of basil plants. Through the application of a multitude of analysis techniques, we observed different responses to BP contamination. Our findings stressed the complexity of BP interactions with basil plants and showed that corn starch-based BP has the potential to severely impact crop plants. However, it can be stated that concentrations above 1.5% (*w*/*w*) of bioplastic in soil represent threshold values of toxicity to plants, as a significant decrease in yield parameters and an increase in oxidative and osmotic stress in the aerial part of basil plants was observed, which significantly impaired the physiological developmental performance of plants. Overall, these findings emphasize the need for further research to understand the underlying mechanisms and long-term environmental implications of BP pollution on the plant–soil system.

## Figures and Tables

**Table 1:** Effect of different corn starch-based BP concentrations [ranging from 0% (control = C) to 3%, *w*/*w*)] in the soil on macro- (calcium—Ca, potassium—K, magnesium—Mg, phosphorus—P, and sulfur—S), and micronutrient (copper—Cu, iron—Fe, manganese—Mn, sodium—Na, and zinc—Zn) content determined in the shoot of basil plants. Different letters = statistically significant differences.

Nutrient	Unit	C	B0.5	B1	B1.5	B2	B2.5	B3
**Ca**	g kg^−1^	28.2 ± 1.1 ^ab^	25.7 ± 0.3 ^b^	26 ± 1.1 ^b^	27.6 ± 0.6 ^ab^	27.1 ± 0.6 ^ab^	27.2 ± 0.4 ^ab^	28.8 ± 0.8 ^a^
**K**	g kg^−1^	61.4 ± 1.5 ^a^	61 ± 1.6 ^a^	61.2 ± 1.1 ^a^	62.7 ± 0.8 ^a^	66.1 ± 1 ^a^	61.9 ± 4.2 ^a^	51.5 ± 0.4 ^b^
**Mg**	g kg^−1^	5.6 ± 0.3 ^b^	4.9 ± 0.1 ^b^	4.8 ± 0.1 ^b^	4.9 ± 0.5 ^b^	5.5 ± 0.1 ^b^	5.4 ± 0.1 ^b^	6.8 ± 0.2 ^a^
**P**	g kg^−1^	9.9 ± 0.1 ^a^	5.9 ± 0.3 ^b^	4.9 ± 0.3 ^c^	3.1 ± 0.2 ^d^	2.9 ± 0.06 ^d^	2.4 ± 0.2 ^d^	2.6 ± 0.03 ^d^
**S**	g kg^−1^	3.6 ± 0.4 ^a^	3.2 ± 0.1 ^a^	3.9 ± 0.4 ^a^	3.9 ± 0.1 ^a^	3.8 ± 0.1 ^a^	4 ± 0.3 ^a^	3.7 ± 0.2 ^a^
**Cu**	mg kg^−1^	8.20 ± 0.2 ^b^	7.89 ± 0.1 ^b^	8.19 ± 0.5 ^b^	8.79 ± 0.4 ^b^	8.48 ± 0.5 ^b^	7.72 ± 0.5 ^b^	15.38 ± 0.5 ^a^
**Fe**	mg kg^−1^	83.42 ± 2 ^b^	80.21 ± 2 ^b^	81.37 ± 3 ^b^	95.98 ± 11 ^b^	95.48 ± 6 ^b^	93.14 ± 0.9 ^b^	163.35 ± 10 ^a^
**Mn**	mg kg^−1^	96.63 ± 6 ^a^	73.18 ± 1 ^b^	54.47 ± 0.7 ^c^	50.53 ± 3 ^c^	54.54 ± 1 ^c^	55.53 ± 2 ^c^	50.86 ± 1 ^c^
**Na**	mg kg^−1^	475.98 ± 9 ^b^	471.18 ± 47 ^b^	471.57 ± 75 ^b^	476.71 ± 66 ^b^	453.73 ± 47 ^b^	457.07 ± 81 ^b^	684.30 ± 58 ^a^
**Zn**	mg kg^−1^	103.89 ± 5 ^ab^	120.42 ± 8 ^a^	96.94 ± 8 ^b^	105.91 ± 9 ^ab^	92.60 ± 4 ^b^	102.34 ± 6 ^ab^	112.81 ± 6 ^ab^

## Data Availability

The data presented in this study are available on request from the corresponding author.
